# RNA-sequencing reveals altered skeletal muscle contraction, E3 ligases, autophagy, apoptosis, and chaperone expression in patients with critical illness myopathy

**DOI:** 10.1186/s13395-019-0194-1

**Published:** 2019-04-16

**Authors:** Monica Llano-Diez, Wen Fury, Haruka Okamoto, Yu Bai, Jesper Gromada, Lars Larsson

**Affiliations:** 1 Department of Physiology and Pharmacology, Karolinska Institutet, Bioclinicum, J8:30, SE-171 77 Stockholm, Sweden; 2 Regeneron Pharmaceuticals, Inc., Tarrytown, NY 10591 USA; 3 Department of Clinical Neuroscience, Karolinska Institutet and Karolinska Hospital, Stockholm, Sweden

**Keywords:** Critical illness myopathy, RNA sequencing, Skeletal muscle transcriptomics, Gene expression, Intensive care unit, Mechanical loading, Muscle atrophy

## Abstract

**Background:**

Critical illness myopathy (CIM) is associated with severe skeletal muscle wasting and impaired function in intensive care unit (ICU) patients. The mechanisms underlying CIM remain incompletely understood. To elucidate the biological activities occurring at the transcriptional level in the skeletal muscle of ICU patients with CIM, the gene expression profiles, potential upstream regulators, and enrichment pathways were characterized using RNA sequencing (RNA-seq). We also compared the skeletal muscle gene signatures in ICU patients with CIM and genes perturbed by mechanical loading in one leg of the ICU patients, with an aim of reducing the loss of muscle function.

**Methods:**

RNA-seq was used to assess gene expression changes in tibialis anterior skeletal muscle samples from seven critically ill, immobilized, and mechanically ventilated ICU patients with CIM and matched control subjects. We also examined skeletal muscle gene expression for both legs of six ICU patients with CIM, where one leg was mechanically loaded for 10 h/day for an average of 9 days.

**Results:**

In total, 6257 of 17,221 detected genes were differentially expressed (84% upregulated; *p* < 0.05 and fold change ≥ 1.5) in skeletal muscle from ICU patients with CIM when compared to control subjects. The differentially expressed genes were highly associated with gene changes identified in patients with myopathy, sepsis, long-term inactivity, polymyositis, tumor, and repeat exercise resistance. Upstream regulator analysis revealed that the CIM signature could be a result of the activation of MYOD1, p38 MAPK, or treatment with dexamethasone. Passive mechanical loading only reversed expression of 0.74% of the affected genes (46 of 6257 genes).

**Conclusions:**

RNA-seq analysis revealed that the marked muscle atrophy and weakness observed in ICU patients with CIM were associated with the altered expression of genes involved in muscle contraction, newly identified E3 ligases, autophagy and calpain systems, apoptosis, and chaperone expression. In addition, MYOD1, p38 MAPK, and dexamethasone were identified as potential upstream regulators of skeletal muscle gene expression in ICU patients with CIM. Mechanical loading only marginally affected the skeletal muscle transcriptome profiling of ICU patients diagnosed with CIM.

**Electronic supplementary material:**

The online version of this article (10.1186/s13395-019-0194-1) contains supplementary material, which is available to authorized users.

## Background

The ability of the skeletal muscle to sense mechanical stimuli is important for regulation of gene expression and protein synthesis and enables the muscle to adapt to altered physiological demands. The process is referred to as tensegrity [1, 2]. Conversely, loss of tensegrity due to mechanical silencing (absence of external strain related to weight-bearing and internal strain in the muscle fiber caused by myosin-actin activation) causes muscle wasting and weakness. This explains in part why critically ill, immobilized, and mechanically ventilated intensive care unit (ICU) patients develop severe muscle wasting and impaired muscle function [3, 4]. The most frequent primary myopathy causing muscle weakness and paralysis in the ICU is called critical illness myopathy (CIM), which is found in up to 30% of the general ICU population and up to 100% in certain subgroups [5]. Moreover, CIM has been associated with delayed recovery, increased morbidity and financial costs, and impaired quality of life among survivors [6, 7]. CIM is characterized by marked myosin loss, muscle atrophy affecting muscle fibers expressing type I and II myosin heavy chain (MyHC) isoforms, and reduced muscle membrane excitability [5, 8]. The pathophysiology of CIM is complex and remains incompletely understood, but it involves activation of protein degradation pathways, transcriptional downregulation of myofibrillar proteins, decreased membrane excitability, mitochondrial dysfunction, and altered excitation-contraction coupling [9, 10]. Microarray technology has been used to elucidate skeletal muscle gene expression changes in critically ill and mechanically ventilated ICU patients and in animal models mimicking the ICU condition [11–13], but high-throughput RNA sequencing (RNA-seq) studies to identify differentially expressed genes (DEGs) and upstream regulators in ICU patients with CIM are still lacking. The aim of this study was to investigate gene expression changes in skeletal muscle from critically ill and mechanically ventilated ICU patients with CIM to understand the pathophysiological processes occurring in the skeletal muscle from CIM patients and to provide novel insight into the mechanisms underlying muscle weakness in ICU patients.

No specific and effective treatment is available for CIM. Supportive strategies such as early rehabilitation help improve patients’ recovery and functional outcomes. There has been an interest in the study of the effects of passive movement on the skeletal muscle to preserve the architecture of the muscle fibers and prevent protein loss and function in mechanically ventilated and paralyzed critically ill ICU patients [14]. Our previous work showed that passive mechanical loading had positive effects on muscle fiber function in both experimental and clinical studies [15, 16], but the mechanisms underlying this finding remain incompletely understood. Consequently, an additional aim of this study was to explore if preservation of muscle function by mechanical loading for 10 h a day for an average of 9 days on one leg of ICU patients affected by CIM reduced the disease gene signature when compared to the unloaded leg from the same patient.

## Methods

### Patients and control subjects

Percutaneous conchotome muscle biopsies from the tibialis anterior (TA) muscle were used for RNA-seq from seven mechanically ventilated ICU patients (five women aged 41–80 years and two men aged 55 and 68 years) and six control subjects (five women aged 68–82 years and one man aged 72 years). The majority of the patients were neuro-ICU patients (*n* = 4) who were immobilized after neurosurgery, cerebral lesions and infections. The other patients were mechanically ventilated and immobilized due to sepsis after a colon perforation, chronic obstructive pulmonary disease with pneumonia, and thrombocytopenia purpura with neurological and renal complications. All patients had been mechanically ventilated for longer than 2 weeks at the time of muscle biopsy and electrophysiological examination. All patients were diagnosed with CIM based on electrophysiological findings that reflected reduced compound muscle action potentials upon supramaximal stimulation of motor nerves, normal or subnormal motor nerve conduction velocities, and the presence of spontaneous electromyographic activity (typically positive sharp waves and fibrillation potentials) in some of the proximal and distal upper and lower extremity muscle investigated; additionally, patients displayed the hallmark of CIM, i.e., the preferential loss of the motor protein myosin when normalized to actin content [10, 17]. Myosin to actin ratios were measured as previously described [15]. The average myosin to actin ratios in patients with CIM were 0.65 ± 0.43 (all patients had a myosin to actin ratio below 1.5). In a separate study, muscle biopsies were simultaneously obtained bilaterally from the TA in seven mechanically ventilated neuro-ICU patients, where one leg was subjected to mechanical loading for 2.5 h, four times a day for 9 ± 1 days. The donor characteristics and the mechanical loading procedure have been described [15]. The muscle biopsies were obtained using the percutaneous conchotome method on the final day of the observation period. Each biopsy was dissected and treated as previously described [15, 18]. Written informed consent was obtained from patients’ close relatives prior to the study. The study was approved by the Ethical Committee on Human Research at Karolinska Institutet, Stockholm and Uppsala University Hospital, Uppsala, Sweden.

### Transcriptome sequencing data generation

The protocols for RNA extraction and sequencing library preparation were similar to those described earlier [19]. The RNA samples were treated with DNase prior to RNA-seq using the RNase-free DNase kit according to the manufacturer’s instructions (Qiagen, Inc., Valencia, CA). An Advanced Analytical Fragment Analyzer was used for RNA quality analysis, and the RNA quality number (RQN) is included in Additional file 1. Strand-specific RNA-seq libraries were prepared from 500-ng RNA using the KAPA stranded mRNA-Seq Kit (KAPA Biosystems, Wilmington, MA, USA). The libraries were amplified by 12-cycle PCR. Sequencing was performed on an Illumina HiSeq®2000 (Illumina) by multiplexed single-read run with 33 cycles. The resulting FASTQ files were analyzed via FastQC to ensure sufficient data quality. The reads were mapped to the human genome (hg19) using commercial software ArrayStudio (OmicSoft) with two mismatches allowed. The average reads sequenced was about 16.8 million per sample and the average reads aligned to transcripts was about 97.0%. Among them, 83–90% reads were uniquely mapped.

### Differential gene expression analysis

Gene expression was quantified by the number of reads mapped to the sense-strand exons and converted to reads per kilobase per million (RPKM). Genes were flagged as detectable with an empirical minimum RPKM of 0.1. For comparison between two groups of samples, genes were eliminated if they were not detectable in the number of samples that is greater or equal to the smaller sample size of the two comparing groups. Fold changes were computed for the remaining genes as the ratio between the arithmetic mean RPKM values of the two groups. The statistical significance of the differential expression was assessed by Student’s *t* test. Genes with a fold change ≥ 1.5 in either direction and with a *p* < 0.05 were reported as significantly differentially expressed genes (DEGs). False discovery rate (FDR) was also calculated and provided, even though the values were not used as part of the threshold to obtain significant genes.

Gene Ontology (GO) biological process analysis was performed with the PANTHER Overrepresentation Test (released 2017205) in PANTHER™ version 13.1 (http://www.pantherdb.org/). The topmost significantly differentially regulated 1266 genes were used; these genes were ranked by absolute fold change after meeting the thresholds of *p* < 0.05 and fold change ≥ 3.0, as determined by Fisher’s exact test with the FDR multiple test correction. In addition, some GO categorization was performed manually (independent of PANTHER) to improve the interpretative value of the data.

### Pathway enrichment analysis

The resulting DEG signatures were compared with the canonical pathways in MSigDB. The statistical significance of the overlap between individual pathways with a given list of gene signatures was evaluated by NextBio Running Fisher test. Pathways were ranked by their significance, and the 15 top pathways were reported herein.

### Ingenuity upstream regulator and pathway analysis

The topmost significantly perturbed skeletal muscle genes in CIM patients when compared to healthy control subjects (*t* test *p* < 0.05 and fold change > 5 in either direction) were subjected to Ingenuity Upstream Analysis (IPA, QIAGEN Redwood City, www.qiagen.com/ingenuity). The most significant upstream analysis results were reported based on *p* < 1.0E−4 and activation *z*-score > 2. Genes differentially expressed in the passively exercised leg and the nonloaded control legs (*t* test *p* < 0.05 and fold change > 1.5 in either direction) were also analyzed by Ingenuity Canonical Pathway analysis.

## Results

### Gene signature of skeletal muscle from ICU patients with CIM

**Fig. 1:**
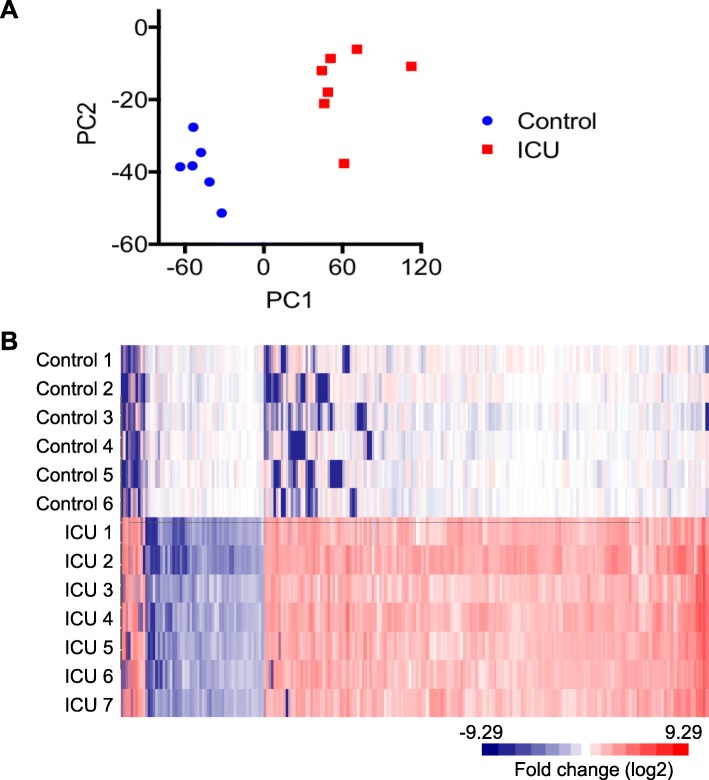
PCA and heat map of DEGs of muscle samples from ICU patients and healthy controls. **a** PCA of TA skeletal muscle samples from healthy control subjects and ICU patients with CIM using all genes (RPKM log2 transformed and mean centered) **b** Heat map of top 1266 DEGs between ICU patients with CIM and healthy control muscle samples (independent group *t* test *p* < 0.05 and fold difference ≥ 3). RPKM of each gene was normalized against the mean of healthy controls and followed by log2 transformation

We performed transcriptome profiling on the TA skeletal muscles isolated from seven critically ill patients who had been mechanically ventilated in the ICU for more than 2 weeks and six healthy control subjects. We detected 17,221 expressed genes when counting genes with RPKM over 0.1 in at least 6 out of the 13 samples. Figure 1a shows that the skeletal muscle samples from the ICU patients, and corresponding healthy control subjects were well separated into distinct clusters in principal component analysis (PCA) using all genes detected. When comparing the skeletal muscle gene signatures of the ICU patients with CIM to the healthy controls, we found 6257 DEGs (*p* < 0.05 and fold change ≥ 1.5) (Additional file 2). Most of the affected genes were upregulated (84% or 5237 genes), and only 16% (1020 genes) were downregulated. The heat map in Fig. 1b shows very similar changes in the expression of the top 1266 differentially expressed genes (*p* < 0.05 and fold difference ≥ 3) among the ICU muscle samples when normalized against the mean of the healthy controls. Table 1 shows the 15 most significant results of the NextBio correlation analysis. The DEGs in the patients with CIM were highly associated with gene changes identified in patients with myopathy, sepsis, long-term inactivity, polymyositis, tumor, and repeated resistance exercise. Collectively, these data show that the expression of 36% of all detected genes was affected in TA skeletal muscle from patients with CIM and that the gene signatures associated highly with changes were identified in patients with diseases that either directly or indirectly affect skeletal muscle mass and function.

**Table 1:** NextBio correlation analysis of genes perturbed in the muscle of ICU patients. DEGs between ICU patients with CIM and healthy control muscle samples were used as input. The top 15 most significant results are listed

			Comparisons		Correlations to this study		
Project ID	Sample	Species	Bioset 1	Bioset 2	*p* value	Direction	Number of common genes
GSE3307	Skeletal muscle	*Homo sapiens*	Acute quadriplegic myopathy patients	Healthy subjects	6.00E−87	+	2607
GSE13205	Vastus lateralis	*Homo sapiens*	Septic patients with organ failure	Healthy subjects	1.00E−73	+	687
GSE38680	Biceps	*Homo sapiens*	Infantile-onset Pompe patients	Healthy subjects	1.50E−68	+	2779
GSE24215	Vastus lateralis	*Homo sapiens*	9 days of inactivity	Baseline	2.80E−54	+	840
GSE26852	Skeletal muscle	*Homo sapiens*	Polymyositis patients	Healthy subjects	7.20E−51	+	1148
GSE17679	Skeletal muscle	*Homo sapiens*	Ewing’s sarcoma recurrent tumor	Normal tissue	8.10E−50	+	3980
GSE17679	Skeletal muscle	*Homo sapiens*	Ewing’s sarcoma metastasis	Normal tissue	5.40E−46	+	4225
GSE17679	Skeletal muscle	*Homo sapiens*	Ewing’s sarcoma primary tumor	Normal tissue	5.50E−46	+	4451
GSE45426	Vastus lateralis	*Homo sapiens*	3 days of repeat resistance exercise	Baseline	6.60E−44	+	2212
GSE17503	Paravertebral muscle cells	*Homo sapiens*	Cultured	Biopsy without culture	1.90E−42	+	2045
GSE38012	Vastus lateralis	*Homo sapiens*	Subjects on long-term 30% calorie restricted diet	Subjects on Western diet	1.10E−41	−	3239
GSE14901	Vastus lateralis	*Homo sapiens*	14 days of limb immobilization by cast	Baseline	1.60E−41	+	1274
GSE1551	Muscle	*Homo sapiens*	Dermatomyositis patients	Healthy subjects	8.90E−40	+	1239
GSE4411	Tibialis anterior muscles	*Mus musculus*	3 days of denervation	No denervation	9.60E−40	+	1748
GSE26852	Skeletal muscle	*Homo sapiens*	Dermatomyositis patients	Healthy subjects	4.80E−39	+	1027

To obtain more detailed gene expression patterns, selected DEGs associated with GO biological process categorization are discussed below (for fold change and *p* values, please see Additional file 2).

#### Muscle contraction and system process

The software program PANTHER identified “muscle contraction” and “muscle system process” as the most significantly enriched GO terms (fold enrichment 2.89 and 3.05, FDR 3.65E−03 and 1.52E−04, respectively) for the top 1266 DEGs with fold change ≥ 3 and *p* < 0.05. Within this category and when focusing on skeletal muscle contraction, the gene encoding the β/slow type I MyHC isoform (*MYH7*), the dominant isoform in TA muscle, was downregulated. Myosin light chains (*MYL-1, 2 ,3*, and *MYLK2*), actinin-3 (*ACTN3*), myotilin (*MYOT*), and structural sarcomeric proteins such as myomesin-2 (*MYOM2*) were also downregulated. The loss of myosin and myosin-associated proteins is a hallmark of CIM, and the results at the transcriptional level agree with clinical and experimental CIM studies [4, 12, 15]. Actin (*ACTA1*) expression was downregulated in patients with CIM, although the myosin to actin ratios decreased at the protein level. Myogenin (*MYOG*), myogenic factor (*MYF6*), calmodulin-1 (*CALM1*), dystrophia myotonica protein kinase (*DMPK*), and several subunits of acetylcholine receptor (*CHRN*-*A1*, *B1*, *D*, *G*) and sodium channel (*SCN-9A*, *5A, 3B*) were highly upregulated in skeletal muscle of patients with CIM, whereas nitric oxide synthase 1 (*NOS1*) was downregulated.

Out of the top 1266 DEGs, myosin binding protein H (*MYBPH*), tropomodulin-1 (*TMOD1),* and some subunits of dihydropyridine-sensitive L-Type (*CACN-B4*, *B1, G8*) were upregulated, whereas, myosin binding protein C1 (*MYBPC1*), tropomyosin (*TPM3*), and troponin T1 (*TNNI1*) were downregulated. Moreover, genes that play a critical role in skeletal muscle development and regeneration were upregulated, such as the metalloprotease-14 (*MMP1*4) and the transcription factor *SOX6*.

#### Ubiquitin-proteasome system (UPS)

Many genes involved in UPS, such as the proteasome family (PSM), ubiquitin-conjugating enzymes (UBEs), and E3 ligases, were altered in response to the ICU intervention. The two well-studied E3 ubiquitin ligases, *MURF1/TRIM63* and *ATROGIN-1/FBXO32*, showed upregulation, but they did not pass the *p* value cutoff. The recently discovered E3 ligases *FBXO30*/*MUSA1* and *TRIM32* were upregulated, whereas *FBXO40* expression was reduced.

#### Autophagy-lysosome system (ALS)

The cathepsins (*CTSS*, *CTSB*, *CTSA*, *CTSD*, *CTSZ,* and *LGMN*), which are lysosomal proteases, were upregulated, despite the upregulation of several cathepsin inhibitors. The microtubule-associated protein 1 light chain 3 beta (*MAP1LC3B*), *p62*/*SQSTM1*, and the nutrient-deprivation autophagy factor-1 (*NAF1*) had increased expression *(MAP1LC3B* did not pass the p value cutoff). The transcriptional upregulation of these genes is important during the activation of autophagy [20].

#### Calpain system

The expression of calpains, nonlysosomal calcium-dependent proteases, changed in CIM patients. *CAPN-1*, *2*, *10*, and *CAPNS1* were upregulated, while *CAPN6*, *CAPN8*, and the muscle-specific isoform, *CAPN3*, were downregulated.

#### Protein folding and heat shock proteins/chaperones

Proteins with chaperone functions are important for the control of protein folding and the reduction of misfolded protein aggregates. There was an upregulation of a large number of chaperones at the RNA level in patients with CIM; the key endoplasmic reticulum chaperones, calnexin (*CANX*) and calreticulin (*CALR*), tubulin-specific chaperones (*TBCA* and *TBCC*), members of the torsin family (TOR1A, *TOR2A*, and *TOR3A*), and several members of the TCP-1 ring complex (TRiC, also called chaperonin containing TCP-1 [CCT]) were upregulated. The myosin chaperone *UNC-45B*, regulating myosin folding, assembly, and function, was upregulated together with heat shock protein-90 (*HSP90AA1*, *HSP90AB1*, and *HSP90AB4P*). *HSP70* (*HSPA2*) and *HSP20* (*HSPB6*) expression was downregulated in ICU patients with CIM.

#### Apoptosis

The effector caspases, *CASP4* and *CASP6*, which execute apoptosis were upregulated, as were other genes that regulate apoptosis, such as programmed cell death 6 (*PDCD6*), apoptosis antagonizing transcription factor (*AATF*), apoptotic chromatin condensation inducer 1 (*ACIN1*), apoptosis inducing factor mitochondria associated 1 (*AIFM1*), apoptotic peptidase activating factor 1 (*APAF1*), makorin ring finger protein 1 (*MKRN1*), BCL2 associated X apoptosis regulator (*BAX*), heat shock protein beta1 (*HSPB1*), and the E3 ubiquitin ligase *RBX1*. *TP53* (*p53*) and its regulated genes, such as *CDKN1A* (*p21*), *GADD45A*, *PIDD*, *BAX*, and *DDB2* (*p48*), were also upregulated. Apoptosis can also be induced through the activation of death receptors, including tumor necrosis factor (TNF family), which was altered in patients with CIM.

#### mRNA translation

The eIF2 initiation complex regulates mRNA translation. Several genes of this pathway, including EIF2 members (*EIF2*, *EIF2AK4*, *EIF2B4*, *EIF2B2*, *EIF2S2*), many EIF3, EIF4, and EIF5 complex members, and ribosomal subunits, were upregulated, among other genes involved in the pathway, which suggests an activation of muscle protein synthesis in patients with CIM.

### MYOD1, p38 MAPK, and dexamethasone were identified as potential upstream regulators of skeletal muscle gene expression in ICU patients with CIM

**Fig. 2:**
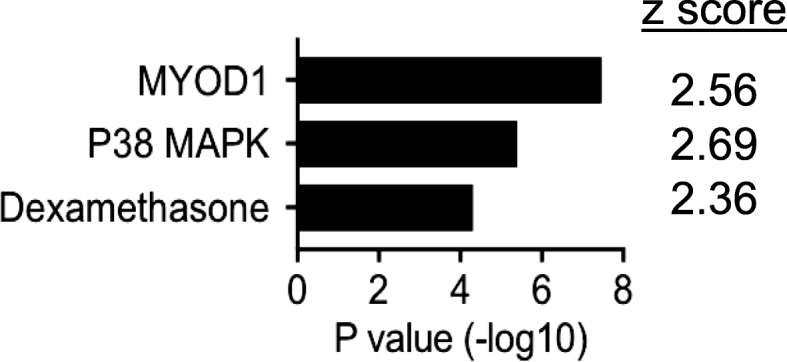
Ingenuity upstream regulator analysis of top skeletal muscle genes perturbed in ICU patients with CIM. The most significantly perturbed genes in the ICU patients with CIM (361 genes, Student *t* test *p* < 0.05 and fold change > 5 in either directions) were subjected to the analysis. Inclusion criteria were *p* < 1.0E−04 and activation *z*-score > 2

Ingenuity upstream regulator analysis was employed to study 361 genes, and a set of top differentially regulated genes among the ICU muscle samples were identified by the following criteria: *p* < 0.05 and fold change > 5. The analysis examines the enrichment of the selected genes in known pathways downstream of a regulator and predicts the direction of activity change (activation *z*-score) for the upstream regulator in relation to what is expected from the literature. We applied cutoff criteria of *p* < 1.0E−4 and the absolute value of activation *z*-score > 2 for either direction. The analysis revealed that genes controlled by MYOD1, p38 MAPK, and dexamethasone were affected in the ICU patients with CIM. These regulators have established roles in skeletal muscle development and regeneration, as well as in response to changes in muscle size [21–24]. They had positive *z*-scores, indicating that activation of MYOD1, p38 MAPK, or the application of dexamethasone could cause gene expression changes similar to those observed in these patients (Fig. 2).

### Effects of passive mechanical loading on skeletal muscle gene signature in ICU patients with CIM

**Fig. 3:**
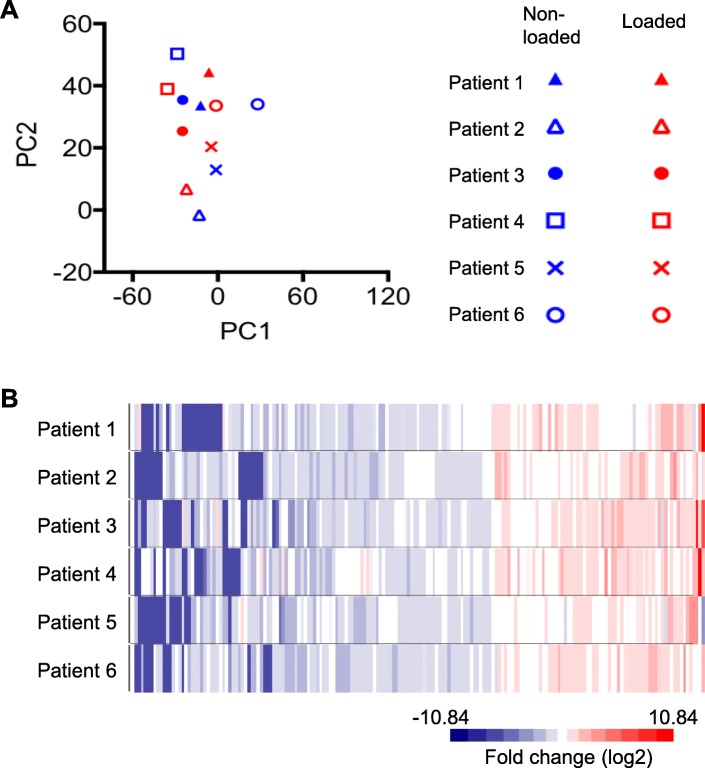
PCA and heat map of DEGs of CIM muscle samples with or without passive loading. **a** PCA of skeletal muscle samples from passively loaded left and nonloaded right legs of ICU patients with CIM using all genes detected (RPKM log2 transformed and mean centered) **b** Heat map of 182 DEGs between muscle samples obtained from passive loaded left and nonloaded right leg muscles of critically ill ICU patients with CIM (dependent group *t* test *p* < 0.05 and fold difference ≥ 1.5). RPKM of each gene was normalized against the nonloaded control of the same patient and followed by log2 transformation

**Fig. 4:**
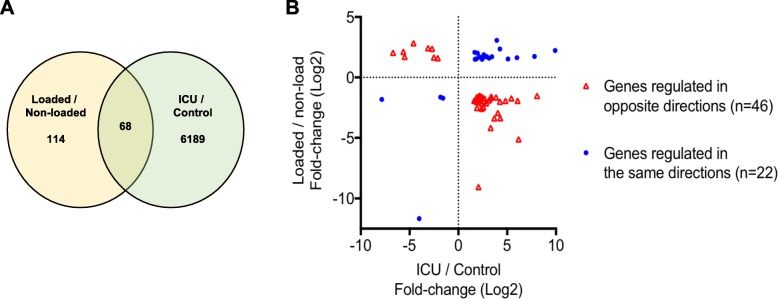
Venn diagram and fold change scatter plot of genes perturbed by CIM or passive loading. **a** Venn diagram of two gene lists: DEGs between passively loaded left and nonloaded right legs of ICU patients with CIM on the left and DEGs between healthy and CIM skeletal muscle samples on the right. **b.** Fold change scatter plot of overlap genes. The opposite effect of the CIM state and passive loading on 46 out of 68 overlap genes is indicated in red (Fisher’s exact test *p* < 0.046)

**Fig. 5:**
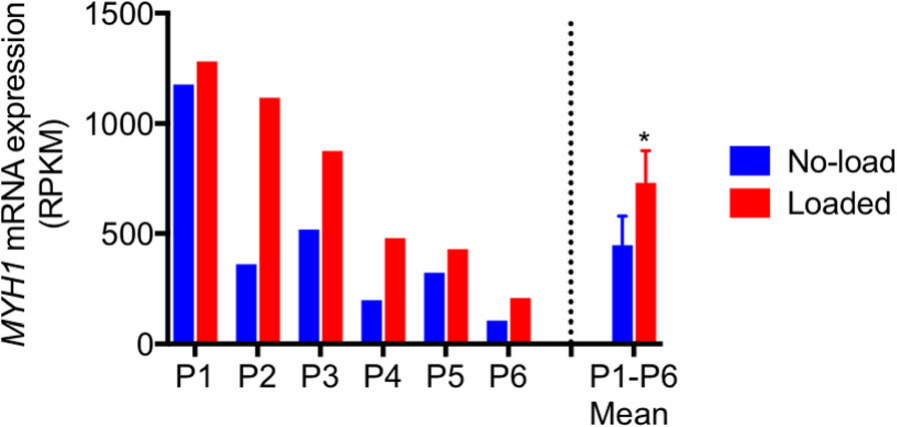
*MYH1* expression in muscle samples in different conditions. Significant increase of *MYH1* expression in the ICU patient muscle samples and further increase in the loaded left compared to the nonloaded right legs

Next, we obtained gene signatures from TA skeletal muscle samples from both legs of six critically ill and immobilized ICU patients who developed CIM; one leg (left) was subjected to passive mechanical loading for 10 h daily for an average of 9 days. In this analysis, we identified 16,594 genes, which were detected (RPKM > 0.1) in at least 6 of the 12 samples. PCA did not reveal a separation of the unloaded and loaded muscle samples (Fig. 3a). Passive mechanical loading of the left leg was previously reported to increase the specific force of single muscle fibers by 35%, but it did not prevent the loss of myosin and muscle mass [25]. It was therefore surprising that we only detected 182 DEGs between muscle samples obtained from passive loaded left and unloaded right leg muscles of the patients with CIM (dependent group *t* test *p* < 0.05 and fold difference ≥ 1.5) (Additional file 3). The heat map in Fig. 3b shows a fairly even expression of the 182 affected genes among patients when their expression was normalized to the unloaded control of the same patient. Ingenuity pathway analysis suggests that these genes were enriched in Phospholipase C Signaling (*p* = 5.48E−3) and Role of NFAT in Immune Response (9.01E−03). Only 68 of the 182 genes were in common with the 6257 affected genes in the patients with CIM (Fig. 4a). Of these 68 genes, 46 were regulated in the opposite direction, and 22 genes were regulated in the same direction (Additional file 4 and Fig. 4). Of the 46 genes regulated in the opposite direction, the expression of 38 genes was upregulated, and 8 were downregulated in the CIM state. The opposite effect of CIM state and mechanical loading on these overlap genes are statistically significant (Fisher’s exact test, *p* = 0.046), suggesting the possible positive effect of mechanical loading in these patients. Interestingly, we found that *MYHC-IIX*/*D* (*MYH1*) expression was sixfold higher in the muscle of ICU patients (Additional file 2), and it was further upregulated by mechanical loading (Fig. 5 and Additional file 3). Collectively, these and previous data show that mechanical loading of legs of ICU patients with CIM improves the specific force of single TA skeletal muscle fibers, despite a preservation of myosin loss and muscle wasting [15], and it reverses the expression of only 46 genes out of 6257 affected genes in the ICU patients with CIM.

We did not compare the samples from the two studies. The principal component analysis (PCA) suggested that samples from the two studies were separated. The lack of overlap between the CIM samples from the two studies could potentially be due to differences in procedures for sample acquisition, treatment, and handling.

## Discussion

CIM is a potentially lethal condition, affecting more than 30% of the ICU population and up to 100% in certain subgroups [5]. However, the gene expression and molecular pathways influencing skeletal muscle wasting and weakness in ICU patients with CIM are incompletely known. To our knowledge, this is the first time that RNA-seq was used to elucidate the skeletal muscle transcriptome profiling and upstream regulators of critically ill, immobilized, and mechanically ventilated ICU patients with CIM. Moreover, the effects of passive mechanical loading on the skeletal muscle transcriptome signature of patients with CIM have also been studied. Compared with traditional technologies to study gene expression, such as microarrays, high-throughput RNA-seq provides unprecedented sensitivity and reproducibility to discover DEGs while showing high accuracy for quantifying expression levels and closely correlating with quantitative PCR and RNA spike-in control samples [26, 27].

Skeletal muscle adapts readily to environmental factors, nutritional interventions, loading conditions, and contractile activity by altering muscle fiber size, functional capacity, and metabolism. It has been demonstrated that mechanical silencing is a significant factor triggering the severe muscle atrophy and impaired function associated with CIM [15, 28], and the present results show a profound impact at the skeletal muscle transcriptional level in ICU patients with CIM. Out of 17,221 detected genes, 6257 (36%) were affected in TA skeletal muscle from the patients with CIM. The expression of most of the genes was upregulated and revealed a high degree of similarity to the gene signatures available for patients with myopathy, polymyositis, sepsis, cancer, and immobilization, and the gene changes correlated best with gene signatures from patients with myopathy, immobilization, sepsis, polymyositis, and cancer. Ingenuity upstream regulator analysis revealed that genes regulated by MYOD1, p38 MAPK, and dexamethasone treatment are affected in the ICU patients with CIM. These molecules have established roles in skeletal muscle development and regeneration, as well as in response to cellular stress. MYOD1 is a transcription factor that regulates muscle cell differentiation and skeletal muscle regeneration and plasticity in response and hypertrophic or atrophic conditions [21, 22, 29, 30]. P38 MAPK is involved in skeletal muscle differentiation and development and in cast-immobilized atrophy via MuRF1 [23, 31]. Dexamethasone is a glucocorticoid that can induce skeletal muscle atrophy and myosin loss associated with upregulation of MuRF1 and atrogin-1 and downregulation of HDAC6 [32, 33].

The three proteolytic systems (UPS, ALS, and the calcium-dependent calpains) were activated in CIM. In addition to the upregulation (although not statistically significant) of *MURF1* and *ATROGIN-1*, two newly identified and critical E3 ligases that control muscle size, *TRIM32* and *FBXO30/MUSA1*, were upregulated in response to muscle atrophy associated with CIM. TRIM32 binds to myosin and ubiquitinates desmin, as well as thin filament (actin, tropomyosin, troponins) and Z-band (α-actinin) proteins [34]. TRIM32 expression is not upregulated under all forms of atrophy, and its role in the regulation of muscle mass is still not clear [35]. Moreover, TRIM32 knockout mice develop atrophy and show impaired muscle regrowth after atrophy [36]. FBXO30/MUSA1 is upregulated by denervation-induced atrophy, and the knockdown of MUSA1 reduces muscle atrophy after denervation [37]. FBXO40 is upregulated during muscle differentiation, and FBXO40 knockout mice show increased muscle size [38]. *FBXO40* expression was downregulated in patients with CIM. This result is reminiscent of findings in dystrophic muscles of limb-girdle muscular dystrophy (LGMD) patients and starvation-related muscle atrophy, but it contrasts with an upregulation in response to denervation-induced muscle atrophy [39].

Autophagy can be activated by starvation, exercise, critical illness, and a variety of stress signals [20]. Autophagy may contribute to muscle protein degradation by increased breakdown of damaged proteins/organelles or may protect myofiber integrity and muscle tissue homeostasis by clearing abnormal organelles and by sequestering cytotoxic products that result from myofibrillar protein degradation [40]. Recently, a considerable number of studies have confirmed a protective role of autophagy against various types of organ failure in critically ill animal models [41]. Several cathepsins, *MAP1LC3B*, which has an essential role in the formation of autophagosomes, and *p62/SQSTM1*, which mediates the breakdown of protein aggregates by binding to LC3 and polyubiquitinated proteins [42], had increased expression in our study. In addition, *NAF1*, a Bcl-2 associated autophagy regulator that is essential for the maintenance of skeletal muscle mass [43], was also upregulated. This indicates that activation of ALS plays an important role in patients with CIM, although its detrimental vs. protective roles need to be further studied.

The calpain system, which disassembles myofibrillar proteins from the sarcomere to be ubiquitinated and degraded, is activated in the ICU patients with CIM. The expression of the muscle-specific isoform *CAPN3* decreased, in accordance with findings in animal models of CIM [12] and denervation-induced atrophy and regeneration [44]. However, CAPN3 is primarily involved in sarcomere remodeling and does not contribute to the increased protein degradation.

Altered chaperone expression has been reported in a porcine model of critical illness [45]. *HSPB6* (α-crystallin-related) expression was downregulated in CIM patients, in accordance with our results [12]. HSPB6 plays several roles in skeletal muscle contractile machinery and is associated with troponin complexes in the protection from muscle atrophy, ischemia, hypertensive stress, and metabolic dysfunction [46]. UNC-45 is a critical chaperone for myosin folding, stability, and function and protects myosin from stress [47]. In the current study, *UNC-45B* expression was upregulated. Overexpression of UNC-45 in muscle cells results in increased myosin degradation and reduced or disorganized myofibrils [48]. Moreover, loss or overexpression of UNC-45 leads to defective myofibril organization in the skeletal muscle of zebrafish embryos [49]. UNC-45 binds to HSP90, acting as a cochaperone in myosin folding and protection against stress [47]. *HSP90* expression was upregulated in parallel with *UNC-45B* in the studied patients with CIM. Therefore, the higher expression of both HSP90 and UNC45 results in disassembly of the myosin filament. HSP70 is a key chaperone family involved in the maintenance of muscle fiber integrity and regulation of muscle regeneration [50]. *HSP70* expression was downregulated in ICU patients with CIM, contrary to rodent models of CIM [12]. Conversely, HSP70 expression is reduced during muscle inactivity and aging, leading to muscle atrophy and impaired function [50].

Apoptosis is implicated in the development of muscle atrophy induced by several conditions, i.e., disuse or aging, and is needed in the maintenance of skeletal muscle homeostasis [51, 52]. Apoptosis is orchestrated by caspases and activated by cell cycle regulators, particularly those that induced cell death, and proapoptotic genes such as *PDCD6*, *AATF*, *ACIN1*, *AIFM1*, *APAF1*, *MKRN1*, and *BAX*, which were upregulated in the ICU patients with CIM; additionally, the caspases *CASP4* and *CASP6* and several genes related to the p53 pathway were activated. Activation of apoptosis at the mRNA level and increased myonuclear apoptosis have been previously reported in an experimental ICU model [12] in accordance with the results obtained from the ICU patients diagnosed with CIM.

Sarcomeric protein gene expression was significantly affected by CIM. The downregulation of MyHC isoforms and actin at the transcriptional level, despite decreased myosin to actin ratios at the protein level, has been previously reported during the acute phase of CIM [3]. This indicates that protein synthesis is affected. The differences between gene and protein expression might be due to posttranscriptional regulation, protein degradation, or protein turnover rate, as actin has a twofold longer protein turnover rate than myosin [3, 53]. We have previously seen an upregulation of *MYBPH* in a rat model of CIM [12] and in ICU patients with CIM during recovery [3], suggesting a role of *MYBPH* in the maintenance and reassembly of the thick filament structure in patients with CIM [3].

The muscle protein translational machinery was activated in accordance with our previous observations in an experimental ICU model [12, 28]. In this context, it is interesting to note that stimulation of muscle protein synthesis signaling pathways has also been reported in critically ill ICU patients [54], despite marked muscle wasting, suggesting that protein degradation was exceeding the synthesis rate. The increased protein synthesis has been suggested to be related to an increased availability of amino acids from the increased proteolysis and/or an attempt to counteract muscle mass loss [54]. Moreover, the activation of several genes that play a critical role in skeletal muscle development and regeneration, such as myogenic factors (*MYOG*, *MYF6*) [55]; metalloproteinase *MMP14* [56]; the transcription factor *SOX6*, which also regulates muscle fiber type differentiation [57, 58]; and *DMPK*, whose reduction also contributes to muscle wasting in muscular dystrophies [59], together with the transcriptional upregulation of genes that participate in the translational machinery, may indicate compensatory mechanisms to reduce the excessive sarcomeric degradation and muscle wasting seen under ICU conditions.

Surprisingly, passive mechanical loading for 10 h a day for 9 ± 1 days only marginally affected the skeletal muscle gene signature of another group of CIM patients. Single muscle fiber contractile measurements from the same samples were previously shown to increase specific force by 35% in response to passive loading [15]. These results could indicate that the improvement in specific muscle fiber force occurs primarily at the post-transcriptional level, but mass-spectrometry analyses of myosin post-translational modifications (PTMs) did not show any significant differences between the loaded and the unloaded leg [15]. Other PTMs in thin filament proteins may be affected by the loading condition, with important consequences for the regulation of muscle contraction. In addition, it is important to emphasize that the present gene expression data reflect the average gene changes in the skeletal muscle tissue, whereas the specific force measurements were obtained from single fibers with sufficient integrity to undergo contractile measurements. Interestingly, the expression of *MyHC-IIx/d* (*MYH1*) increased 500% in the CIM state (Additional file 2) and further increased 64% in the loaded leg at the transcriptional level (Fig. 5 and Additional file 3), but these increases could not counteract the loss of myosin and muscle mass [15]. There is a slow turnover of myosin in skeletal muscle, and one cannot exclude that the initial preferential myosin loss targets a pool of newly synthesized myosin or myosin destined for degradation (that is, myosin not involved in force generation). In previous experimental studies, we have shown that activation of different protein synthesis and degradation pathways in response to mechanical ventilation and immobilization follow a strict temporal pattern [12, 28, 60]. For logistic reasons, all gene expression measurements in this study were conducted at one late time point during the unilateral mechanical loading in immobilized and mechanically ventilated ICU patients. Therefore, it cannot be excluded that early activation of mechanosensitive pathways that preceded the time of muscle biopsy collection have gone undetected in the current study. In an attempt to improve our understanding of the temporal gene expression pattern in ICU patients developing CIM, we are presently following a group of ICU patients with six repeated muscle biopsies during 12 days of mechanical ventilation and immobilization. Another potential explanation for the small difference in gene expression between loaded and unloaded leg is the crossover effects on gene expression via circulating factors released from the loaded muscles, such as myokines or exosomes. Based on our previous experimental work, we do no not find any experimental evidence of a crossover effect by unilateral mechanical loading [19, 61]. However, recent experimental findings from our group show a strong crossover effect in response to unilateral direct muscle electrical stimulation, presumably generated via calcium-stimulated exosome release mediating muscle-to-muscle communication (in preparation).

## Conclusions

Mechanical silencing in critically ill ICU patients who developed CIM is associated with profound changes in skeletal muscle gene expression. RNA-seq analysis revealed that the marked muscle atrophy and weakness seen in ICU patients with CIM were associated with altered expression of genes involved in muscle contraction and system process, newly identified E3 ligases, autophagy and calpain systems, apoptosis, and chaperone expression at the transcriptional level. Ingenuity upstream regulator analysis additionally revealed that the CIM signature is reminiscent of those detected by activation of MYOD1, p38 MAPK, or treatment with dexamethasone. However, mechanical loading only marginally affected the skeletal muscle transcriptome profiling of ICU patients diagnosed with CIM.

## Additional files

Additional file 1: RNA quality number (RQN). (XLSX 13 kb)

Additional file 2: 6257genes_withFDR. (TXT 284 kb)

Additional file 3: 182genes_withFDR. (TXT 8 kb)

Additional file 4: Of these 68 genes, 46 were regulated in the opposite direction, and 22 genes were regulated in the same direction. (XLSX 14 kb)

## Abbreviations

ALS: Autophagy-lysosome system
CIM: Critical illness myopathy
DEGs: Differentially expressed genes
GO: Gene Ontology
HSP: Heat shock protein
ICU: Intensive care unit
MyHC: Myosin heavy chain
PCA: Principal component analysis
PTMs: Post-translational modifications
RNA-seq: RNA sequencing
RPKM: Reads per kilobase per million
TA: Tibialis anterior
UBEs: Ubiquitin-conjugating enzymes
UPS: Ubiquitin proteasome system
